# Zero Ischemia Open Partial Nephrectomy in Crossed Fused Ectopic Kidney - A Rare but Real Challenge

**DOI:** 10.7759/cureus.44011

**Published:** 2023-08-23

**Authors:** Deerush Kannan, Sindhu Sankaran, Madhav Tiwari, Sandeep Bafna, Narasimhan Ragavan

**Affiliations:** 1 Urology, Apollo Hospitals, Chennai, IND; 2 Urology, Addenbrooke's Hospital, Cambridge University Hospitals NHS Foundation Trust, Cambridge, GBR

**Keywords:** zero ischemia, congenital anomaly of kidney and urinary tract, open partial nephrectomy, crossed fused ectopic kidney, renal cancer

## Abstract

Crossed fused renal ectopia (CFRE) is a rare congenital anomaly with both kidneys located on the same side of the retroperitoneal space. Due to complex anatomy, any renal tumours arising from this congenital anomaly will require careful pre-operative planning and intra-operating management to ensure oncological clearance while maximizing renal function. In this clinical case, a 57-year-old lady was referred to our center with a left to right CFRE and a 10cmx8cmx8cm mass arising from the interpolar region of left ectopic kidney on a background of multiple medical co-morbidities including stage 3a chronic kidney disease (CKD). Careful pre-operative planning and optimization was done, including 3D reconstruction of CT images, and the decision was made to perform a zero ischemia open partial nephrectomy to give her kidneys the best fighting chance. She recovered well postoperatively with only a mild increase in creatinine and histopathology revealing a renal cell carcinoma. The case emphasizes the need for adequate pre-operative planning with the use of upcoming imaging modalities like 3D reconstruction for optimum planning to ensure the best postoperative outcomes.

## Introduction

Crossed fused renal ectopia (CFRE) is a congenital anomaly very rarely encountered wherein both kidneys are located on the same side of the retroperitoneal space [1]. The incidence of CFRE cases varies from 1 in 2000 to 1 in 7500 [2,3]. This condition has a varied presentation ranging from incidental detection to renal impairment. Considering the rarity of the condition, very few cases with a renal tumor arising from CFRE have been reported in the literature. Herein, we bring an interesting rare case of zero ischemia open partial nephrectomy for a renal cell carcinoma arising from the ectopic kidney in a left-to-right CFRE.

## Case presentation

A 57-year-old lady with right lower abdomen pain was referred to our tertiary care center as the contrast CT of the abdomen and pelvis suggested a left to right crossed fused renal ectopia (CFRE) with a mass from the fused left kidney. Her past medical history included diabetes, hypertension, hypothyroidism, and chronic kidney disease (grade 3a, as classified by Kidney Disease Improving Global Outcomes or KDIGO). She could do her regular activities to manage her lifestyle, and her World Health Organization (WHO) performance status was grade 1. Blood workup showed a Haemoglobin of 8.1 gm/dl, creatinine (1.3 mg/dL), and estimated glomerular filtration rate (eGFRö 55.3 mL/min/1.73 m) with the other parameters within range. Contrast CT revealed left to right CFRE with a malrotated right renal hilum facing anterolaterally and left renal hilum facing anteriorly. There was a large (10x8x8 cm) well-defined round peripherally calcified non-enhancing hypo to iso dense (10 to 60 HU) lesion noted in the interpole region of ectopic left kidney causing splaying of renal vessel and the calyceal system and indenting the upper pole of right kidney with the lesion showing few enhancing internal septations and no pelvicalyceal system (PCS) dilatation.

**Figure 1:**
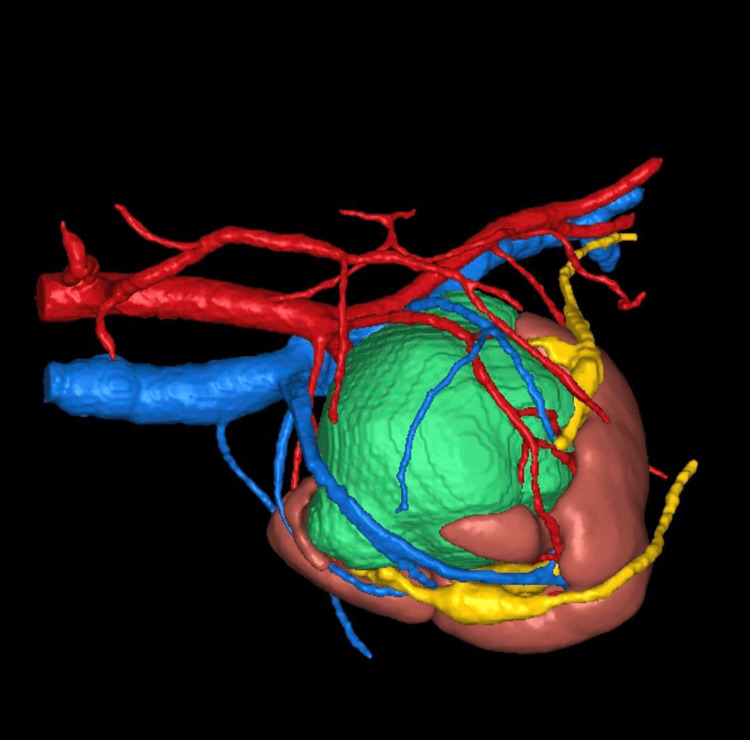
3D reconstruction image of tumor in crossed fused ectopic kidney

A second opinion was sought from the in-house radiologist, and 3D reconstruction of the kidney (Figure 1) with its vasculature was performed by Innersight laboratories, UK. Since the CT features were suspicious of malignancy and it had complex vascular anatomy with the mass being large in size (around 10 cm), after discussion in the Pan India tumor board meeting, we offered the option of open partial nephrectomy. The patient and her family decided to proceed with an open partial nephrectomy.

After performing the cystoscopy, which showed a normal position of bilateral ureteric orifices and retrograde pyelogram again demonstrating the left PCS on the right side inferior to the left PCS, stenting of bilateral ureters was performed in a way to facilitate the identification and prevent intra-operative ureteric injuries as the mass was in close proximity to the ureter and splaying the calyceal system. This was followed by a lower midline laparotomy to perform an open partial nephrectomy (PN) by a transperitoneal approach. Intraoperatively, as reported by the CT, a large mass of 10 cm in the maximum dimension was appreciable, arising from the posterior aspect of the fused left kidney. The 3D reconstruction helped us in safe dissection around the mass as we had an idea about the proximity of the vasculature, especially while dissecting the tumor to expose its posterior most aspect that was very close to the iliac vessels.

**Figure 2:**
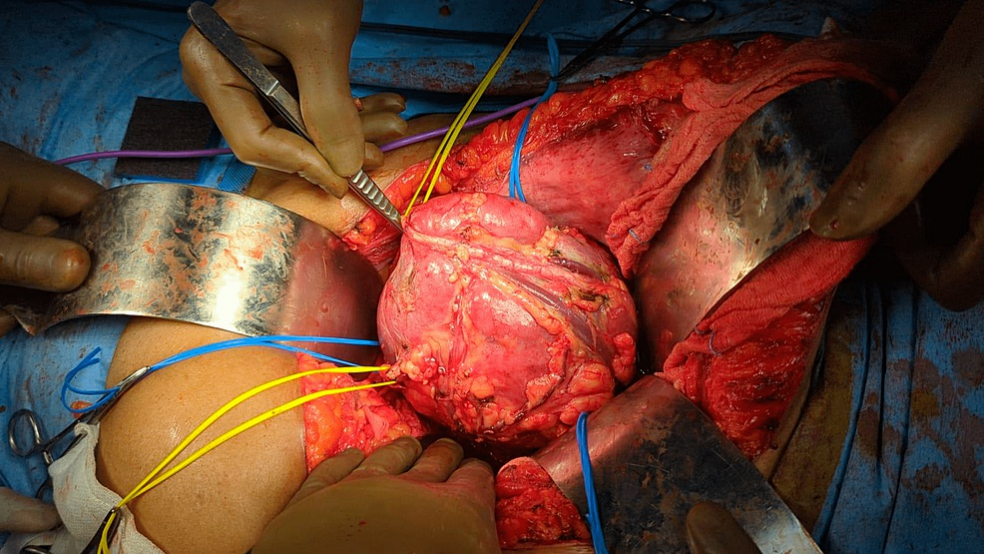
Intraoperative image

There were three arterial supplies, with one arising from the aorta and two arteries arising from the right common iliac artery to supply the fused kidney. A similar pattern was the venous drainage with tributaries draining into the inferior vena cava (IVC) and the right common iliac vein. All the supplying arteries and veins were looped independently (Figure 2). The tumor was delineated on all sides (the posterior aspect was visualized clearly after flipping the kidney), and the margins were marked with monopolar cautery. In view of multiple vessels supplying the tumor and the caliber of the vessels being smaller than that of a normal renal artery, zero ischemia resection was performed in the enucleation plane as the vasculature was not consistent for clamping. In addition to that, the precise vascular supply to the tumor was not distinctly clear as the tumor was very central and hilar in location. Resection was performed, and midway through the resection.

One of the vascular supplies had to be ligated to achieve clearance; however, this did not lead to the devascularisation of the kidney, and the kidney remained pink throughout the procedure. Bleeding was controlled using multiple suture ligation with absorbable sutures, and second-layer renorrhaphy was performed by sliding clip renorrhaphy technique. After the completion of the procedure, adequate time was taken for observing the kidney's vascularity and urine output, ensuring the kidney complex was well perfused and urine output was adequate. Subsequent to that, a drain was placed in the pararenal area. The peritoneum and Gerota were partially closed in order to secure the kidney in the retroperitoneum (nephropexy). The abdomen was closed in layers.

Postoperatively, the patient was monitored in the surgical high-dependency unit for a day, had a ward stay for four days, and was discharged from the hospital on the fifth postoperative day (POD) without any complications. She had a higher drain output for two days that settled subsequently. Creatinine increased from normal to 2.0 mg/dl and started settling down by day five. Foley was removed on POD seven, and the drain was removed on POD 10. The histopathology arrived as renal cell carcinoma (pT2a N0), with margins being negative.

## Discussion

Congenital renal fusion anomalies are characterized by partial or complete fusion of the two kidneys, and they can present in the form of horseshoe kidney, CFRE, and fused pelvic kidney. CFRE is one of the rarest anomalies of the genito-urinary system, wherein both kidneys are located on one side of the midline and are fused with each other [1]. Most cases remain asymptomatic during life and are diagnosed incidentally.

Ectopia with fusion is more common than its counterparts without fusion. Among the reported cases, it is inferred that the condition is more common in males, and left-to-right ectopia with an inferior fusion of the contralateral kidney is referred to as the most common variety [4]. This statement holds true in our case except for the fact the patient was female otherwise with a left to right ectopia with inferior fusion.

Apart from the present case, 18 cases of renal cell carcinoma (RCC) have been reported in the literature in patients with CFRE. The most common histopathologic finding was a clear cell variant that was reported in 10 out of the 18 cases (56%). The clinical presentations in these patients were similar to the age-old description of the presentation of RCC patients, including a loin or back pain associated with haematuria and an abdominal mass. There have only been four cases of PN for RCC in patients with CFRE to date. The first-ever case of PN in CFRE was reported by Sugita et al., and a transperitoneal approach to the kidney was successfully attempted [5]. Since it was a very large mass, we decided to perform PN with a transperitoneal incision. In two of the four operated cases, ureteric stents were deployed prior to PN. We, too, had a strong belief that stent placement would facilitate intra-operative identification of the ureters, especially in such rare instances of anomalies where there can be more surprises intraoperatively, and also it is safer to have stents, especially if there is a breach in PCS (which happened in our case) while resecting such large tumours.

Though Yamamichi et al. reported a case of RCC in a patient horseshoe kidney treated by robotic-assisted partial nephrectomy (RAPN), it could be argued that why couldn't we consider RAPN in cases of RCC with CFRE in experienced hands [6]. We opted for open PN because of the size of the tumor, location, and complex vascularity, and that could not be dealt with even in higher centers and in experienced hands with RAPN.

## Conclusions

This is a very rare tumor, and it needs teamwork along with adequate preoperative planning. We prefer bilateral stenting prior to the procedure as we don't know about the course of the ureters. Vasculature delineation with patient dissection is one of the critical steps, and 3D reconstruction can help in anticipating the course of the vessels, which are highly variable in these cases. The key is to identify every artery and vein and keep them looped independently so that there is an opportunity to get control in case of torrential bleeding. The vessels are tiny, unlike the caliber of normal renal artery, and very easily, these small vessels can go for spasm during dissection. So whenever possible, it is wise to attempt zero ischemia resection owing to these reasons. Slow and gentle step-by-step resection of the tumor and using clamps at the base of resection to control bleeding is recommended. Post-resection papaverine was given locally in our case to ensure flow is good before confirming hemostasis. Waiting for adequate time to look for perfusion of the kidney and thereby confirming hemostasis and urine output is advisable prior to closure.
